# Cross-Sectional and Cumulative Longitudinal Central Nervous System Penetration Effectiveness Scores Are Not Associated With Neurocognitive Impairment in a Well Treated Aging Human Immunodeficiency Virus-Positive Population in Switzerland

**DOI:** 10.1093/ofid/ofz277

**Published:** 2019-07-08

**Authors:** Galia M A Santos, Isabella Locatelli, Mélanie Métral, Alexandra Calmy, Thanh Doco Lecompte, Isaure Nadin, Christoph Hauser, Alexia Cusini, Barbara Hasse, Helen Kovari, Philip Tarr, Marcel Stoeckle, Christoph Fux, Caroline Di Benedetto, Patrick Schmid, Katharine E A Darling, Renaud Du Pasquier, Matthias Cavassini, Neurocognitive Assessment in the Metabolic and Aging Cohort (NAMACO) Study Group

**Affiliations:** 1 Infectious Diseases Service, Lausanne University Hospital, Switzerland; 2 Division of Biostatistics and Quantitative Methods, Institute of Social and Preventive Medicine, Lausanne University Hospital, Switzerland; 3 Laboratory of Neuroimmunology, Research Centre of Clinical Neurosciences, Department of Clinical Neurosciences, Lausanne University Hospital, Switzerland; 4 Service of Neurology, Department of Clinical Neurosciences, Lausanne University Hospital, Switzerland; 5 HIV Unit, Infectious Diseases Division, Department of Medicine, University Hospital of Geneva, Switzerland; 6 Department of Neurology, University Hospital of Geneva, Switzerland; 7 Department of Infectious Diseases, Bern University Hospital, University of Bern, Switzerland; 8 Department of Infectious Diseases and Hospital Epidemiology, Universitätsspital Zurich, Switzerland; 9 University Department of Medicine, Kantonsspital Bruderholz, University of Basel, Switzerland; 10 Infectious Diseases and Hospital Epidemiology Department, Universitätsspital Basel, Switzerland; 11 Infectious Diseases and Hospital Epidemiology Department, Kantonsspital Aarau, Switzerland; 12 Infectious Diseases Division, Ospedale Regionale di Lugano, Switzerland; 13 Infectious Diseases and Hospital Epidemiology Division, Kantonsspital St. Gallen, Switzerland

**Keywords:** aging, cognitive disorders, CPE score, HIV, neurocognitive impairment

## Abstract

**Background:**

Neurocognitive impairment (NCI) in people with human immunodeficiency virus (PWH) remains a concern despite potent antiretroviral therapy (ART). Higher central nervous system (CNS) penetration effectiveness (CPE) scores have been associated with better CNS human immunodeficiency virus (HIV) replication control, but the association between CPE and NCI remains controversial.

**Methods:**

The Neurocognitive Assessment in the Metabolic and Aging Cohort (NAMACO) study is a subgroup of the Swiss HIV Cohort Study (SHCS) that invited patients aged ≥45 years enrolled in the SHCS and followed-up at NAMACO-affiliated centers in Switzerland to participate between May 2013 and November 2016. In total, 981 patients were enrolled, all of whom underwent standardized neurocognitive assessment. Neurocognitive impairment, if present, was characterized using Frascati criteria. The CPE scores of NAMACO study participants with undetectable plasma HIV-ribonucleic acid at enrollment (909 patients) were analyzed. Cross-sectional CPE scores (at neurocognitive assessment) were examined as potential predictors of NCI in multivariate logistic regression models. The analysis was then repeated taking CPE as a cumulative score (summarizing CPE scores from ART initiation to the time of neurocognitive assessment).

**Results:**

Most patients were male (80%) and Caucasian (92%). Neurocognitive impairment was present in 40%: 27% with HIV-associated NCI (mostly asymptomatic neurocognitive impairment), and 13% with NCI related to other factors. None of the CPE scores, neither cross-sectional nor cumulative, was statistically significantly associated with NCI.

**Conclusions:**

In this large cohort of aviremic PWH, we observed no association between NCI, whether HIV-associated or related to other factors, and CPE score, whether cross-sectional or cumulative.

## Teaser

Neurocognitive impairment (NCI) presents a potential disease burden as people living with HIV age on antiretroviral therapy (ART). This study examined the association between ART Central nervous system (CNS) Penetration Effectiveness (CPE) scores and NCI in 909 patients with undetectable plasma viral loads, first by taking cross-sectional CPE scores (single measurement) and then by taking cumulative CPE scores (summary of measurements throughout the entire duration of ART exposure).

Neurocognitive impairment (NCI) in people with human immunodeficiency virus (PWH) remains a concern, even in the era of potent antiretroviral therapy (ART) [1, 2]. Neurocognitive impairment prevalence varies between 25% and 70% [2–4], depending on the patient cohort profile and NCI definition applied. The NCI-associated burden on quality of life, driving capacity, and professional work has been well described [5], and it is likely to increase as the population of PWH grows older. Neurocognitive impairment has been shown to be associated with reduced ART adherence and loss of virological control [6]. In the pre-ART era, NCI was associated with HIV replication in the central nervous system (CNS) and with profound immune suppression [7]. With the widespread use of ART, enabling patients to achieve viral suppression in the plasma and immune recovery, the characteristics of NCI have changed. Although the severe form of NCI according to the Antinori et al [1] definition, human immunodeficiency virus (HIV)-associated dementia (HAD), is less common, the prevalence of less severe forms of NCI, particularly asymptomatic NCI (ANI), has increased [8], and NCI prevalence overall remains high [9]. To explain this phenomenon, factors beyond plasma viral suppression have been explored. These include persistent low-grade chronic immune activation [10] and aging comorbidities such as metabolic syndrome [11], which may be responsible for CNS vascular disorders. Psychiatric disorders including depression, which are common in PWH, may contribute to cognitive impairment [12]. Although a possible association between hepatitis C virus coinfection and risk of NCI remains controversial [12, 13], other factors cited as associated with NCI include female gender, low education level, unemployment, substance misuse, anemia, and thrombocytopenia [12]. Central nervous system viral escape and cerebrospinal fluid (CSF) HIV replication have been reported as possible causes of NCI [14, 15]. Indeed, one study reported that almost 10% of patients with undetectable plasma HIV-ribonucleic acid (RNA) had detectable virus in the CSF [16]. It has also been hypothesized that low-level viral replication in the CNS may contribute to persistent immune activation [10]. Antiretroviral drugs with good CNS penetration may better control replication in this compartment and reduce the risk of developing NCI. Letendre et al [17, 18] defined a CNS penetration effectiveness (CPE) score, based on the physicochemical properties, pharmacokinetics, and pharmacodynamics of each antiretroviral agent prescribed. The CPE score gives an indication of each agent’s capacity to penetrate the CNS and inhibit viral replication. A high CPE score has been associated with a decrease in CSF HIV-RNA [17, 19], and increasing the CPE score of patients’ ART regimens has been related to improved HIV replication control in the CNS [15, 20]. Nevertheless, the association between CPE score and NCI remains controversial [21]. Some authors [22–27] have reported lower NCI frequency in individuals with higher CPE scores. Other studies have found no association between CPE scores and NCI [2, 28] or even a negative association [29, 30]. Neurotoxicity of some ART agents has been proposed as a pathological mechanism underlying such a negative association [31]. To our knowledge, studies to date of associations between CPE score and NCI use the CPE score as a cross-sectional variable. The potential association between NCI and a cumulative CPE score, representing the entire longitudinal history of a patient’s ART exposure over years, has not been explored. The aim of this study was to focus on patients with undetectable plasma HIV-RNA and to examine (1) the relationship between NCI and cross-sectional CPE scores (CPE score at the time of neurocognitive assessment) and then (2) the longitudinal relationship between NCI and cumulative CPE scores (a summary of CPE scores from the time of ART initiation to the time of neurocognitive assessment). Because cognitive impairment may develop progressively during years of HIV infection and ART exposure, we hypothesized that the cumulative CPE score might better predict the presence of NCI than the cross-sectional CPE score.

## METHODS

### Neurocognitive Assessment in the Metabolic and Aging Cohort Study

The Neurocognitive Assessment in the Metabolic and Aging Cohort (NAMACO) is an ongoing prospective longitudinal observational substudy of the Swiss HIV Cohort Study ([SHCS] www.shcs.ch) [32]. The aim of the NAMACO study is to examine the cognitive and neurological impact of HIV infection on an aging HIV-positive population. Patients aged ≥45 years old, enrolled in the SHCS, followed up at 7 cantonal university-affiliated hospital centers, and able to understand neuropsychologists’ instructions in 1 of the 4 languages used (French, German, Italian, and English, translation of neuropsychological [NP] tests resulting from expert consensus) were invited to participate between May 1, 2013 and November 30, 2016, resulting in 981 NAMACO study participants. A standardized neurocognitive assessment was performed by neuropsychologists on all participants at inclusion.

**Figure 1.**
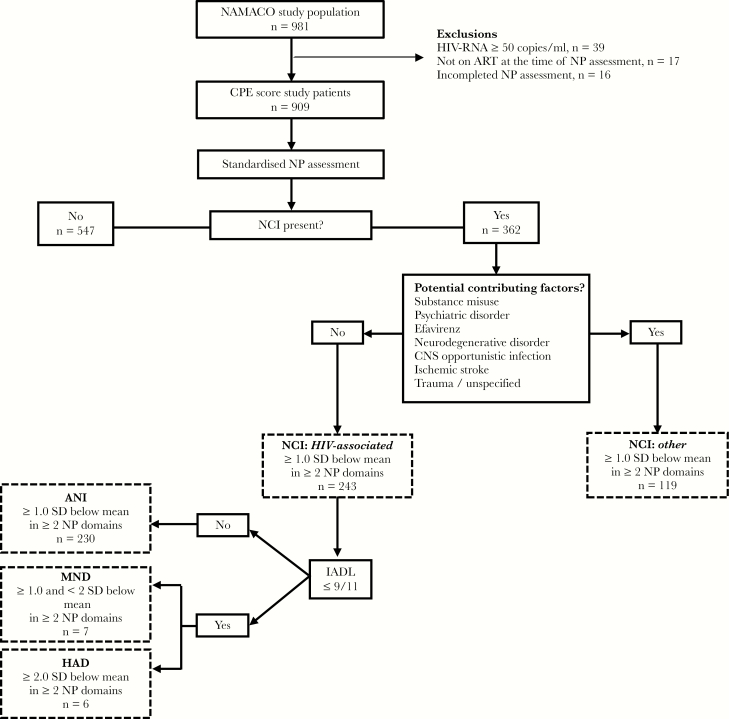
Flowchart of inclusion and assessment of patients in this study from the Neurocognitive Assessment in the Metabolic and Aging Cohort (NAMACO) study population. The nosology of neurocognitive impairment (NCI) is defined using the Frascati criteria [1]. ANI, asymptomatic neurocognitive impairment; ART, antiretroviral therapy; CD, cognitive domain; CES-D, Center for Epidemiological Studies Depression scale; CNS, central nervous system; CPE, CNS penetration effectiveness; HAD, human immunodeficiency virus (HIV)-associated dementia; IADL, Lawton’s Instrumental Activities of Daily Living with 3 supplementary questions; MND, mild neurocognitive disorder; NP, neuropsychological; SD, standard deviation of normative data.

In this study on CPE scores, we excluded patients who were viremic (plasma HIV-RNA ≥50 copies/mL), who were not on ART therapy at inclusion, or whose neurocognitive assessment was incomplete, leaving 909 patients (Figure 1). Human immunodeficiency virus physicians in Switzerland have a free choice when prescribing ART for their patients and base this choice on the European AIDS Clinical Society guidelines, the recommendations of the International AIDS Society USA, or those of the US Department of Health and Human Sciences [33].

Analyses were performed on cross-sectional and retrospective data from the SHCS database, collected at twice-yearly standardized SHCS clinical visits, and data at inclusion into the NAMACO study. The ethics committees of participating centers approved the protocol. All participants provided written informed consent.

### Neurocognitive Assessment

The standardized neurocognitive test battery covered 7 cognitive domains involving the following NP tests: Hopkins Verbal Learning Test-Revised (assessing verbal learning and memory), WAIS-IV Digit Span (attention and working memory), Color Trails Tests (speed of information processing, executive functions), WAIS-IV Digit Symbol subtest (speed of information processing), Victoria Stroop Test (executive functions, language, sensory, and perceptual skills), 5-point Figural Fluency (executive functions), Category Fluency (executive functions, language), Grooved Pegboard dominant and nondominant hand test (motor skills, sensory, and perceptual skills), and Finger Tapping dominant and nondominant hand test (motor skills). The raw score for each NP test was converted to a demographically adjusted standard score (z-score). To quantify the impact of NCI, if present, on functional state, Lawton’s Instrumental Activities of Daily Living (IADL) were assessed with 3 supplementary questions on professional work quality and productivity [9] and on the comments of patients’ relatives regarding cognitive decline, inspired by the Patients Assessment of Own Functioning Inventory questionnaire [34]. Functional impairment was defined as the report of difficulties in at least 2 items of 11. Depressive symptoms severity was graded using the Center for Epidemiologic Studies Depression (CES-D) scale. In this study, individuals with scores ≥27 were considered to suffer from severe depression potentially contributing to NCI. We adopted a cutoff of ≥27, rather than of ≥16 (which would include patients with moderate to severe low mood), to have stringent criteria for non-HIV-associated NCI. Because neurocognitive assessment was performed by trained neuropsychologists, their clinical judgment was applied when categorizing NCI, for example, for patients with CES-D scores between 16 and 26 in whom NCI was considered to be related more to depression than to HIV.

### Neurocognitive Impairment Definition

The revised American Academy of Neurology criteria [1] were used to classify the profile of NCIs into 5 categories: normal neurocognitive assessment, ANI, mild neurocognitive disorder (MND), HAD, and non-HIV-associated NCI (“other”). According to Frascati criteria [1], ANI and MND cases were defined as a decrease of at least 1 standard deviation below normative data in at least 2 cognitive domains without (ANI) or with (MND) functional impairment. Human immunodeficiency virus-associated dementia was defined as a decrease of at least 2 standard deviations in 2 cognitive domains with functional impairment. Non-HIV-associated NCI described individuals with abnormal neurocognitive assessment and at least 1 factor other than HIV infection potentially contributing to cognitive impairment. These factors included substance use, psychiatric disorders including depression, and structural damage associated with previous opportunistic CNS infection, neurodegenerative disorder, or stroke (Figure 1).

### Central Nervous System Penetration Effectiveness Score

The revised CPE score [18] was calculated for each ART agent (for newer agents, unpublished CPE ranks were personally communicated by Dr. Scott Letendre [35] [written communication, 22 June 2018]) (Table 1). Each agent was attributed a rank between zero (absence of CNS penetration) and 4 (highest possible CNS penetration). Each ART regimen CPE score was calculated as the sum of the CPE scores of each agent included in the regimen. Cobicistat and ritonavir as pharmacological boosters were assigned a CPE score of zero.

**Table 1.** Central Nervous System Penetration Effectiveness Scores for Antiretroviral Agents Used to Date

0	1	2	3	4
Amprenavir	Tenofovir disoproxil fumarate	Didanosine	Abacavir	Zidovudine
Amprenavir/r	Tenofovir alafenamide^a^	Lamivudine	Emtricitabin	Nevirapine
Cobicistat	Zalcitabine	Stavudine	Efavirenz	Dolutegravir^a^
	Enfuvirtide	Etravirine	Delavirdine	Indinavir/r
	Ritonavir^b^	Rilpivirine^a^	Raltegravir	
	Saquinavir	Elvitegravir^a^	Maraviroc	
	Saquinavir/r	Fos-amprenavir	Fos-amprenavir/r	
	Tipranavir/r	Atazanavir	Indinavir	
	Nelfinavir	Atazanavir/r	Lopinavir/r	
			Darunavir/r	

NOTE: Adapted from Letendre et al [18] with permission.

^a^Letendre et al [35] with permission and personal communication from Dr. Scott Letendre (unpublished data), written communication (22 June 2018).

^b^When used as a nonbooster.

### Cross-Sectional Central Nervous System Penetration Effectiveness Score

Cross-sectional CPE score at the time of neurocognitive assessment was analyzed continuously, after dichotomization (high ≥7 versus low <7 CPE scores) [36] and after categorization (low ≤5, medium 6–8 and high ≥9 CPE scores).

### Cumulative Central Nervous System Penetration Effectiveness Score

After examining the effect of cross-sectional CPE scores on NCI, we then examined CPE as a cumulative score. Every day of ART was attributed a CPE score according to the regimen prescribed at the time. On this basis, 3 models expressing the cumulative CPE score were used to assess a potential association with the presence of NCI. In the first model, the sum of all CPE scores of every treatment day were calculated and divided by the total time elapsed from ART initiation (mean daily CPE score). In the second model, each day of treatment was classified according to a threshold CPE score of 7. The effect of the percentage of days spent with a high CPE score ≥7 versus a low CPE score <7 on NCI was tested. In the third model, a 3-level categorization based on the CPE score distribution was explored (≤5, 6–8, ≥9). The relationship between the percentages of days spent with a CPE score ≤5 and ≥9 and cognitive dysfunction was explored in the same model. The third model allowed testing of the hypothesis that both high and low CPE scores might be deleterious to cognitive function.

### Socio-Demographics, Human Immunodeficiency Virus Infection, and Comorbidities

Adjustment covariables for multivariate analyses extracted from the SHCS database included the following: age, sex, origin, education level, HIV transmission risk group (men who have sex with men, heterosexual contact, intravenous drug use [IVDU], other), percentage of time with undetectable plasma HIV-RNA (<50 copies/mL), current CD4 cell count, nadir CD4 cell count (<200, ≥200 cells/μL), time since initiation of ART, current efavirenz prescription, hemoglobin, platelet count, CES-D scale score, current cannabis and/or cocaine use, past and/or current IVDU, hepatitis B and/or C virus seropositivity, syphilis seropositivity, diabetes mellitus, arterial hypertension, history of cardiovascular disease (myocardial infarction, ischemic stroke, cerebral hemorrhage, coronary angioplasty and/or angioplasty of peripheral arteries), and the presence of potential contributing factors (listed in Figure 1).

### Statistical Analysis

Statistical analyses were performed using STATA IC 14 and R. Univariate and multivariate analyses were conducted using logistic regression models. Cross-sectional and cumulative CPE scores were compared in patients with NCI (HIV-associated: ANI, MND, and HAD; and non-HIV-associated: other) and in patients with normal neurocognitive assessment. The models described above (1) analyzed the effect of the cross-sectional CPE score at the time of neurocognitive assessment, as a continuous, as a dichotomized, and as a 3-level categorical variable and (2) tested the continuous and categorized effects of the cumulative CPE score. Unadjusted and adjusted analyses were performed. Thereafter, analyses were repeated with exclusion of patients identified as having non-HIV-associated NCI (other). All multivariate analyses of cumulative CPE scores were adjusted for the CPE score at the time of neurocognitive assessment.

## RESULTS 

### Study Population

The majority of patients were male (80%) and Caucasian (92%). Median age was 53 years and median duration of education was 13 years (Table 2). In all, 39.8% of patients presented NCI: 26.7% (243 of 909) diagnosed with HIV-associated NCI (25.3% ANI), and 13.1% diagnosed with non-HIV-associated NCI related to other factors (Table 2, Supplementary Table S1).

**Table 2.** Neurocognitive Assessment in the Metabolic and Aging Cohort (NAMACO) Study Patient Characteristics and Neurocognitive Classification

Patient Characteristics	NAMACO Study (N = 909)
Age (years), median (IQR)	53 (49–59)
Sex, male n (%)	724 (79.7)
Ethnicity n (%)	
Caucasian	834 (91.8)
Other (Black, Hispanic, Asian)	75 (8.2)
Education (years), median (IQR)	13 (12–14)
HIV Transmission Risk Group n (%)	
Men having sex with men	466 (51.3)
Heterosexual contact	302 (33.2)
Intravenous drug use	73 (8.0)
Other^a^	68 (7.5)
Neurocognitive impairment (NCI), n (%)	
Normal neurocognitive assessment	547 (60.2)
Asymptomatic neurocognitive impairment	230 (25.3)
Mild neurocognitive disorder	7 (0.8)
HIV-associated dementia	6 (0.6)
Other—non-HIV-associated NCI^b^	119 (13.1)

Abbreviations: ART, antiretroviral therapy; CNS, central nervous system; HIV, human immunodeficiency virus; IQR, interquartile range.

^a^Transfusion, perinatal transmission, uncertain cause.

^b^Presence of factors other than HIV infection potentially contributing to neurocognitive impairment: CNS opportunistic infection, ART toxicity, psychiatric disorder, substance use, neurodegenerative disorder, and ischaemic stroke.

### Human Immunodeficiency Virus Infection Characteristics and Central Nervous System Penetration Effectiveness Score

All participants had undetectable plasma HIV-RNA, and 56% had CD4 cell count nadirs <200 cells/μL. The median nadir and current CD4 cell counts were 180 (interquartile range [IQR], 73–268) and 638 (IQR, 473–822) cells/μL, respectively (Table 3). The median time since ART initiation was 12 years (IQR, 6–8) (all patients studied being on ART at inclusion). The cross-sectional CPE score at the time of neurocognitive assessment was ≥7 in 79.2% of cases. The median of the distribution of mean cumulative CPE scores was 6.66 (IQR, 5.67–7.39), and the median percent of days spent with a CPE score of ≥7 since ART initiation was 68% (IQR, 42–98) (Table 3).

**Table 3.** Neurocognitive Assessment in the Metabolic and Aging Cohort (NAMACO) Study Patient HIV Infection Characteristics and Central Nervous System Penetration Effectiveness (CPE) Score

HIV Infection Characteristics and CPE Score	NAMACO Study (N = 909)
Current CD4 cell count (cell/μL) median (IQR)	638 (473–822)
Nadir CD4 cell count (cell/μL)	
Continuous median (IQR)	180 (73–268)
<200, n (%)	510 (56.1)
≥200, n (%)	399 (43.9)
Time since ART initiation (years), median (IQR)	12 (6–18)
ART regimen^c^, n (%)	
Nucleoside reverse-transcriptase inhibitor	868 (95.5)
Nonnucleoside reverse-transcriptase inhibitor	451 (49.6)
Protease inhibitor	395 (43.5)
Integrase inhibitor	238 (26.2)
Entry/fusion inhibitor	19 (2.1)
Cross-Sectional CPE Score	
Continuous median (IQR)	7 (7–8)
<7, n (%)	189 (20.8)
≥7, n (%)	720 (79.2)
Total, n (%)	909 (100.0)
≤5, n (%)	38 (4.1)
6–8, n (%)	654 (72.0)
≥9, n (%)	217 (23.9)
Total, n (%)	909 (100.0)
Cumulative CPE Score (Entire ART Duration)^d^	
Mean daily CPE score, median (IQR)	6.66 (5.67–7.39)
Percent ART days spent with CPE score <7, median (IQR)	31.8 (1.9–58.6)
Percent ART days spent with CPE score ≥7, median (IQR)	68.2 (41.4–98.1)
Percent ART days spent with CPE score ≤5, median (IQR)	15.3 (0.0–38.8)
Percent ART days spent with CPE score 6–8, median (IQR)	57.0 (29.8–93.5)
Percent ART days spent with CPE score ≥9, median (IQR)	8.1 (0.0–36.5)

Abbreviations: ART, antiretroviral therapy; IQR, interquartile range.

^a^At the time of neurocognitive assessment.

^b^Among patients with detectable viremia (≥50 copies/mL).

^c^ART regimen at the time of baseline neurocognitive assessment.

^d^Note that the values expressed are median percentages of days spent with CPE scores within a certain group. In this way, the median percentages calculated when there are more than 2 CPE score groups (that is, in the case of ≤5, 6–8, and ≥9) will not have a final sum of 100%.

### Central Nervous System Penetration Effectiveness Score and Neurocognitive Impairment (NCI): Normal Versus NCI (Asymptomatic NCI, Mild Neurocognitive Disorder, Human Immunodeficiency Virus-Associated Dementia, and Other)

In univariate analyses (Table 4, 1st column), neither cross-sectional nor cumulative CPE scores were significantly associated with the presence of NCI. In multivariate analyses (Table 4, 2nd column), none of the CPE scores analyzed demonstrated a statistically significant association or trend with the presence of NCI. No effect was observed when grouping together the 2 extreme categories (≤5 and ≥9) (Supplementary Table S2).

**Table 4.** Association Between Central Nervous System Penetration Effectiveness (CPE) Score and Neurocognitive Impairment

CPE scores ANI, MND, HAD, Other	Unadjusted			Adjusted^a^		
Cross-sectional analysis (at the time of neurocognitive assessment)						
N	909			900		
	OR	95% CI	*P*	OR	95% CI	*P*
Continuous	1.03	0.96–1.11	.408	1.04	0.94–1.14	.441
≥7	1.13	0.81–1.57	.476	1.22	0.81–1.83	.347
≤5	0.89	0.45–1.75	.735	0.82	0.35–1.92	.646
6–8 (Ref.)	1.0	-	-	1.0	-	-
≥9	1.06	0.77–1.45	.713	1.16	0.80–1.69	.433
Cumulative analysis (entire ART duration)^b^						
N	909			900		
	OR	95% CI	*P*	OR	95% CI	*P*
Continuous/T	1.04	0.95–1.14	.360	1.02	0.89–1.16	.812
≥7/T (%)	1.02	0.98–1.06	.347	1.03	0.97–1.10	.323
≤5/T (%)	0.98	0.93–1.04	.494	0.99	0.90–1.10	.885
≥9/T (%)	1.01	0.96–1.06	.649	1.00	0.94–1.07	.959

Abbreviations: ANI, asymptomatic neurocognitive impairment; ART, antiretroviral therapy; CI, confidence interval; CES-D, Center for Epidemiologic Studies Depression; HAD, human immunodeficiency virus-associated dementia; HIV, human immunodeficiency virus; IV, intravenous; MND, mild neurocognitive disorder; OR, odds ratio; other, non-HIV-associated neurocognitive impairment; Ref., the reference against which other categories were compared; RNA, ribonucleic acid; T, time since ART initiation.

NOTE: Odds ratios for ANI, MND, HAD, and other are shown in part A, and those for ANI, MND, and HAD are shown in part B.

^a^Adjustment variables: age, age [2], sex, ethnicity, education (years), T, T^2^, HIV transmission risk group, nadir CD4 cell count (<200, ≥200 cells/μL), proportion of time spent with plasma HIV-RNA <50 c/mL, hemoglobin (categorical variable, according to sex: < lower limit of reference range, within reference range, > upper limit of reference range), platelet count, diabetes, arterial hypertension, antecedent of cardiovascular events, cannabis consumption, cocaine consumption, past and/or actual IV drug use, CES-D scale, current efavirenz prescription, positive hepatitis C serology, positive hepatitis B serology, positive syphilis serology, CPE score at the time of neurocognitive assessment (only for cumulative CPE score analyses representing the entire ART duration).

^b^Odds ratios related to ≥7/T, ≤5/T, and ≥9/T thresholds express the effect of a 10% increase in the percentage of time spent in the specified category.

**Table 5.** Association Between Central Nervous System Penetration Effectiveness (CPE) Score and Neurocognitive Impairment

CPE scores ANI, MND, HAD	Unadjusted			Adjusted^a^		
Cross-sectional analysis (at the time of neurocognitive assessment)						
N	790			783		
	OR	95% CI	*P*	OR	95% CI	*P*
Continuous	1.04	0.96–1.13	.364	1.05	0.94–1.16	.398
≥7	1.15	0.79–1.67	.477	1.25	0.79–1.96	.338
≤5	0.86	0.39–1.89	.710	0.87	0.34–2.25	.774
6–8 (Ref.)	1.0	-	-	1.0	-	-
≥9	1.11	0.78–1.58	.553	1.21	0.81–1.83	.354
Cumulative analysis (entire ART duration)^b^						
N	790			783		
	OR	95% CI	*P*	OR	95% CI	*P*
Continuous/T	1.09	0.98–1.21	.104	1.00	0.86–1.17	.999
≥7/T (%)	1.04	0.99–1.10	.104	1.04	0.98–1.12	.212
≤5/T (%)	0.96	0.90–1.03	.255	1.01	0.90–1.13	.860
≥9/T (%)	1.02	0.97–1.08	.420	1.00	0.93–1.08	.978

Abbreviations: ANI, asymptomatic neurocognitive impairment; ART, antiretroviral therapy; CI, confidence interval; CES-D, Center for Epidemiologic Studies Depression; HAD, human immunodeficiency virus-associated dementia; HIV, human immunodeficiency virus; IV, intravenous; MND, mild neurocognitive disorder; OR, odds ratio; other, non-HIV-associated neurocognitive impairment; Ref., the reference against which other categories were compared; RNA, ribonucleic acid; T, time since ART initiation.

NOTE: Odds ratios for ANI, MND, HAD, and other are shown in part A, and those for ANI, MND, and HAD are shown in part B.

^a^Adjustment variables: age, age [2], sex, ethnicity, education (years), T, T^2^, HIV transmission risk group, nadir CD4 cell count (<200, ≥200 cells/μL), proportion of time spent with plasma HIV-RNA <50 c/mL, hemoglobin (categorical variable, according to sex: < lower limit of reference range, within reference range, > upper limit of reference range), platelet count, diabetes, arterial hypertension, antecedent of cardiovascular events, cannabis consumption, cocaine consumption, past and/or actual IV drug use, CES-D scale, current efavirenz prescription, positive hepatitis C serology, positive hepatitis B serology, positive syphilis serology, CPE score at the time of neurocognitive assessment (only for cumulative CPE score analyses representing the entire ART duration).

^b^Odds ratios related to ≥7/T, ≤5/T, and ≥9/T thresholds express the effect of a 10% increase in the percentage of time spent in the specified category.

### Central Nervous System Penetration Effectiveness Score and Neurocognitive Impairment (NCI): Normal Versus Human Immunodeficiency Virus-Associated NCI (Asymptomatic NCI, Mild Neurocognitive Disorder, and Human Immunodeficiency Virus-Associated Dementia)

None of the CPE scores tested was statistically significantly associated with the presence of NCI in the subgroup of patients with HIV-associated NCI (Table 5). As in the analysis of the entire NAMACO population, no association was seen when grouping together the 2 extreme CPE score categories (Supplementary Table S2).

## Discussion

In this large cohort of HIV-positive patients with undetectable plasma HIV-RNA on ART, we observed NCI in 39.8%: 26.7% with HIV-associated NCI and 13.1% with NCI related to other factors. We found no association between NCI and CPE score, taking CPE score as a cross-sectional variable or as a cumulative score.

Some studies have reported no association between NCI and CPE score, whether using the original 2008 CPE score system or the revised 2010 version [2, 28]. However, other studies have reported a significant association between NCI and a low CPE score [24–26] or a high one [29, 30]. These discrepancies may be explained by study design, differences in NCI definition, or in patient selection, for example, treatment-naive versus treatment-experienced patients, the proportion with undetectable plasma HIV-RNA, and the prevalence of comorbidities.

Although CPE score is taken to indicate the capacity of ART to penetrate into the CSF, it may not accurately reflect ART concentration and efficacy in the CNS. Shikuma et al [10] designed an alternative score, the antiretroviral monocyte efficacy score, which has been associated with better neurocognitive performance. This score is based on in vitro monocyte efficacy data of antiretroviral agents (effective concentration 50 [EC_50_]) and does not take into account the degree of agent CNS penetration. Shikuma et al [10] reported, in mainly well treated patients, that the monocyte efficacy score was associated with the revised 2010 CPE score system. Further studies on the monocyte efficacy score in association with the CPE score are required to determine the relationship between CSF and brain ART concentrations and monocyte efficacy. Indeed, monocytes and macrophages have been reported to play an important role in HIV encephalitis as a potential reservoir in the CNS and as a source of persistent inflammation in this compartment [10]. In our study, we could not use this score because several frequently used antiretroviral agents still do not have EC_50_ data (including etravirine, darunavir, maraviroc, raltegravir, elvitegravir, and dolutegravir). We were not able to confirm our hypothesis that the cumulative CPE score, representing lifetime ART exposure, would predict the presence of NCI better than the cross-sectional CPE score. Some studies have reported a deleterious effect of high cross-sectional CPE scores on neurocognitive function [29, 30]. In our cohort, analyses of the highest CPE scores (≥9) did not support the hypothesis of ART having a toxic effect on the CNS. In our sample of 909 patients, even a small effect should have been detected. Although prescription bias cannot be excluded (patients with cognitive complaints or objective NCI may have been switched to ART regimens with higher CPE scores by their treating clinicians), adaptation of ART regimens according to CPE score in patients with cognitive dysfunction is not recommended, with the exception of patients with discordant HIV-RNA in the CSF compared with plasma [37, 38]. Finally, although analyses were repeated with and without patients identified as having at least 1 non-HIV cause of NCI, the possibility that some patients had mixed NCI etiology (HIV and non-HIV-associated NCI) cannot be excluded, even with our high CES-D score cutoff (≥27). The CPE score aside, it must be emphasized that ART prescription has been reported to improve neurocognitive outcome among PWH [39], and so the potential risk of no treatment clearly outweighs the risk of developing mild cognitive impairment with long-term therapy. Our study has limitations. First, we included patients with comorbidities that could potentially contribute to the development of NCI. However, a sensitivity analysis excluding patients with non-HIV-associated NCI (mostly psychiatric disorders) did not modify our conclusions. The generalization of our results to the entire population of PWH may not be possible: patients were mostly male and Caucasian in our cohort, and we examined individuals ≥45 years old because we wanted to analyze cognition in a well treated aging population living with HIV (longer duration of ART therapy and longer exposure to HIV replication). Although new methods of diagnosing NCI are now evolving, we used a dichotomized outcome variable based on Frascati criteria to enable comparison with other work in the field. With this approach, the vast majority of HIV-associated NCI was classified as ANI. The functional impairment scale we used (IADL) was 8-point rather than 4-point, with supplementary questions on productivity and with comments from the patients’ entourage, and was used by trained neuropsychologists familiar with this type of assessment. However, functional impairment scales can be relatively insensitive, and this might have affected the numbers of patients classified as ANI rather than moderate NCI. For future analyses, we plan to examine the z-scores of NP tests to avoid potential limitations of the Frascati classification system. The small numbers of patients with MND and HAD bring into question whether our study results should be applied only to individuals with mild NCI. At the time of writing, CSF HIV-RNA viral load has not yet been examined because samples are available for only a limited number of patients who agreed to undergo lumbar puncture. Finally, examining cumulative CPE score is not a validated method, but our aim was to test a novel approach to identify associations between CPE score and NCI. Against these limitations, our study has several strengths. The cumulative CPE score, although not validated, is a novel approach that may better represent patients’ exposure to ART than a cross-sectional snapshot. In our study, the SHCS database provided prospectively collected covariables for multiple adjustments; the sample size is large and data are available for the entire duration of HIV infection. The NAMACO study was performed in a real-life setting, including patients of all ART regimens and with neuropsychologists who categorized NCI using clinical judgment as well as scoring systems. Moreover, to our knowledge, ours is the first study of this size in an exclusively aviremic population, enabling neurocognitive assessment among optimally treated patients.

## Conclusions

In conclusion, neither cross-sectional nor cumulative CPE scores were statistically significantly associated with NCI in our large patient cohort. The NAMACO study patients will be reassessed at 2 and 4 years postinclusion, and the effect of ART regimens, and therefore CPE score, will be examined on the appearance, persistence, or resolution of NCI with time.

## Supplementary Data

Supplementary materials are available at *Open Forum Infectious Diseases* online. Consisting of data provided by the authors to benefit the reader, the posted materials are not copyedited and are the sole responsibility of the authors, so questions or comments should be addressed to the corresponding author.

ofz277_suppl_supplementary_tables
