# Supplementary material for: Cross-Sectional and Cumulative Longitudinal Central Nervous System Penetration Effectiveness Scores Are Not Associated With Neurocognitive Impairment in a Well Treated Aging Human Immunodeficiency Virus-Positive Population in Switzerland
Source: Open Forum Infect Dis. 2019 Jul 8;6(7):ofz277. doi: 10.1093/ofid/ofz277 (PMC6612860; doi:10.1093/ofid/ofz277)
Supplement: ofz277_suppl_supplementary_tables [file ofz277_suppl_supplementary_tables.doc]

**Supplementary Table S1.** Non-HIV-associated neurocognitive impairment (*other*) details among aviremic patients enrolled in the Neurocognitive Assessment in the Metabolic and Aging Cohort (NAMACO) study

|  | **NAMACO Study participants** (N=909) |
| --- | --- |
| Central nervous system opportunistic infection, n (%) | 8 (0.9) |
| Psychiatric disorder (diagnosis, CES-D ≥ 27), n (%) | 75 (8.3) |
| Substance use, n (%) | 24 (2.6) |
| Neurodegenerative disorder, n (%) | 3 (0.3) |
| Other (ischemic stroke, trauma, etc), n (%) | 33 (3.6) |

Abbreviations: CES-D, Center for Epidemiologic Studies Depression scale

**Supplementary Table S2.**

| **Part A  CPE scores** ANI, MND, HAD, Other | **Unadjusted** | | | **Adjusted ^a^** | | |
| --- | --- | --- | --- | --- | --- | --- |
| **Cumulative analysis (entire ART therapy)** **^b^** | | | | | | |
| **N** | 909 | | | 900 | | |
|  | **OR** | **95% CI** | ***P*** | **OR** | **95% CI** | ***P*** |
| **≤ 5 / T & ≥ 9 / T** | 1.00 | 0.96 – 1.04 | 0.925 | 1.00 | 0.94 – 1.06 | 0.984 |
| **Part B CPE scores** ANI, MND, HAD | **Unadjusted** | | | **Adjusted ^a^** | | |
| **Cumulative analysis (entire ART therapy) ^b^** | | | | | | |
| **N** | 790 | | | 783 | | |
|  | **OR** | **95% CI** | ***P*** | **OR** | **95% CI** | ***P*** |
| **≤ 5 / T & ≥ 9 / T** | 1.00 | 0.95 – 1.04 | 0.905 | 1.00 | 0.94 – 1.07 | 0.923 |

Odds ratios for ANI, MND, HAD and *other* are shown in part A and those for ANI, MND and HAD are shown in part B.
^a^ Adjustment variables: age, age^2^, sex, ethnicity, education [years], T, T^2^, HIV transmission risk group, nadir CD4 cell count (< 200, ≥ 200 cells/μl), proportion of time spent with plasma HIV-RNA < 50 c/ml, hemoglobin (categorical variable, according to sex: < lower limit of reference range, within reference range, > upper limit of reference range), platelet count, diabetes, arterial hypertension, antecedent of cardiovascular events, cannabis consumption, cocaine consumption, past and/or actual IV drug use, CES-D scale, current efavirenz prescription, positive hepatitis C serology, positive hepatitis B serology, positive syphilis serology, CPE score at the time of neuropsychological examination *(only for cumulative CPE score analyses representing the entire ART therapy)*.
^b^ Odds ratios related to ≤ 5 / T and ≥ 9 / T thresholds express the effect of a 10% increase in the percentage of time spent in the specified category.

Abbreviations: ANI, Asymptomatic Neurocognitive Impairment; MND, Mild Neurocognitive Disorder; HAD, HIV-Associated Dementia; *Other*: Non-HIV-associated neurocognitive impairment; ART, antiretroviral therapy; T, time since ART initiation.
